# Analysis of Land Use Change Drivers and Simulation of Different Future Scenarios: Taking Shanxi Province of China as an Example

**DOI:** 10.3390/ijerph20021626

**Published:** 2023-01-16

**Authors:** Lintao Liu, Shouchao Yu, Hengjia Zhang, Yong Wang, Chao Liang

**Affiliations:** 1 College of Agronomy and Agricultural Engineering, Liaocheng University, Liaocheng 252059, China; 2 College of Water Conservancy and Hydropower Engineering, Gansu Agricultural University, Lanzhou 730070, China

**Keywords:** land use change, land driving factors, geographical detector, CA-Markov, scenario simulation, Shanxi

## Abstract

This study analyzed change and spatial patterns of land use in Shanxi from 2000 to 2020. The drivers of land use and cover change (LUCC) in cultivated lands, forest lands, grasslands, and rural construction areas were explored from four dimensions, including population, natural environment, location traffic, and economic development. The CA-Markov model was used to simulate the scenarios of natural growth (NG), ecological protection (EP), economic development (ED), food security (FS), ecological protection–economic development (EP-ED), and ecological protection–food security (EP-FS) in 2030. The results indicated that: (1) The conversion to built-up areas primarily dominated the LUCC processes, and their expansion was mainly to the detriment of the cultivated lands and grasslands during 2000–2020. (2) From 2000 to 2020, population, economy, and land productivity were the main factors of LUCC; the interaction of drivers for the increase of cultivated lands, forest lands, grasslands, and rural construction areas showed enhancement. (3) Under the NG, ED, and EP-ED scenarios, the rural construction areas would have increased significantly, while under the FS and EP-FS scenarios, the cultivated lands would only just have increased. These future land use scenarios can inform decision-makers to make sound decisions that balance socio-economic, ecological, and food security benefits.

## 1. Introduction

Land use and cover change (LUCC) is the link between human economic activities and natural ecological processes. LUCC can influence international energy balances and biogeochemical cycles, resulting in global ecosystem services [1] and climatic changes [2,3,4]. Human activities have significantly altered much of the Earth’s surface in recent years [5]. Increasing demands for food, water, and shelter from growing human populations have resulted in large-scale deforestation, expansion of urban construction areas [6], fragmentation and loss of agricultural land [7,8], and accelerated evolution of regional land use patterns globally [9], all of which have inevitably affected changes in and the sustainable development of land use systems [10,11,12]. In addition, the impact of human activities on LUCC has led to irreversible loss of biodiversity [9,13,14], isolation of habitat [15], changes in surface temperature [16], and soil erosion [17,18]. Irrational land use will also severely affect carbon sources [19], carbon sinks [20], habitat integrity [21], and food production [21,22], which in turn will exacerbate tensions between humans and the environment [23]. Consequently, it is essential to quantify LUCC dynamics to address climate change, biodiversity loss, and food security [24].

LUCCs are the result of the interaction of many spatiotemporal scale factors [25]. Identifying the relationship between LUCC and its drivers has become indispensable research in recent years [26,27]. Understanding and revealing the LUCC and its driving forces in different regions are significant to regional socio-economic development regulation, land resource management, and ecological environment protection [28]. Furthermore, by revealing the driving force behind LUCC [29], not only can future land use be predicted [30], but it also provides an accurate scientific foundation for effective, green, and sustainable development of regional land use. The geographical detector can detect the spatial differentiation in land use and the interaction between explanatory factors and analytical variables, thereby revealing the drivers of land use [31,32]. The core of the geographical detector is that when an independent variable has an enormous impact on a variable, its spatial distribution must be similar [32,33]. The advantage of a geographic detector is that it can detect not only numerical and qualitative data [34], but also whether there is an interaction between the two factors and the strength, direction, linearity, or non-linearity of the interaction.

Simulating LUCC is a complex issue that involves physical, environmental, demographic, and socio-economic factors [35]. Currently, the commonly used land use simulation models include ANN-CA [36] and CLUE-S models [37,38], but they hardly simulate the complex interaction and competition between different land use types [39,40,41]. The CA model is a discrete dynamical model that simulates the complex interactions and competition between natural phenomena and land use types [42,43,44]. Although the CA model can excellently simulate land use through the definition of transformation rules, it cannot effectively include the time series of LUCC. As a result, many researchers have introduced the Markov chain to achieve better results for the simulation of LUCC [44,45,46], but fewer studies have been conducted to assess the impact of drivers and constraints on LUCC. Lack of assessment of LUCC impact factors ultimately leads to failure of full compliance and uptime monitoring of the atlas. Previous studies have only examined the drivers of a single LUCC, and few have explored the effects on multiple LUCCs in terms of multiple aspects such as population, natural environment, locational traffic, and economic development. In addition, fewer studies have combined geodetectors with CA-Markov, and scenarios of food security were not considered in future scenario simulations.

To address the above-mentioned problems, this study detected the drivers of cultivated lands, forest lands, grasslands, and rural construction areas in Shanxi from the following four dimensions: population, natural environment, regional transportation, and economic development. The CA-Markov model was then used to simulate the scenarios of natural growth (NG), ecological protection (EP), economic development (ED), food security (FS), ecological protection–economic development (EP-ED), and ecological protection–food security (EP-FS) in 2030, which would provide a basis for efficient and sustainable development of land resources in Shanxi in the future. We hypothesized that the data used in the paper are a true and reliable reflection of the study area. Hence, the objectives of the present study were to determine: (1) how did the spatiotemporal transformation of LUCC develop in Shanxi during 2000–2020? (2) What were the driving forces for LUCC from 2000 to 2020? (3) How will LUCC progress under different land use scenarios in the future?

## 2. Study Area and Data Source

### 2.1. Study Area Description

**Figure 1:**
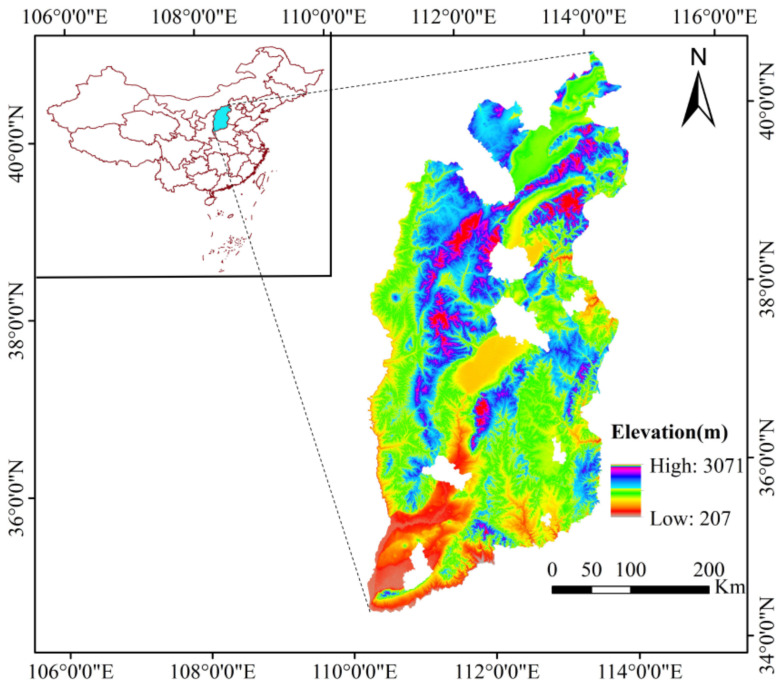
Location of the study area.

Shanxi Province (33°34′–40°44′ N, 110°14′–114°33′ E) is set at an altitude of 207–3071 m and comprises a total area of about 14.1 × 10^4^ km^2^ (Figure 1). The landform types in this area are complex and diverse, and the mountainous area accounts for about one quarter of the total area. The valleys are vertical and horizontal, spanning the two major river systems of the Yellow River and the Haihe River. The overall climate of the region is dry, with average annual temperature of 4.2 to 15.6 °C, in addition to average annual precipitation of 363 to 776 mm. The climate type of Shanxi is classified as a temperate continental monsoon climate zone. The area of ecologically fragile areas is large and the causes of vulnerability are complex. The areas of soil erosion have exceeded 10.8 × 10^4^ km^2^, accounting for 69.5% of the total land area. The areas of extremely sensitive and highly sensitive areas have reached 7.7 × 10^4^ km^2^, accounting for 49.5% of the total land area. The ecological environment in this area has become one of the most critically fragile areas of the world due to soil erosion and environmental degradation.

### 2.2. Data Sources

**Figure 2:**
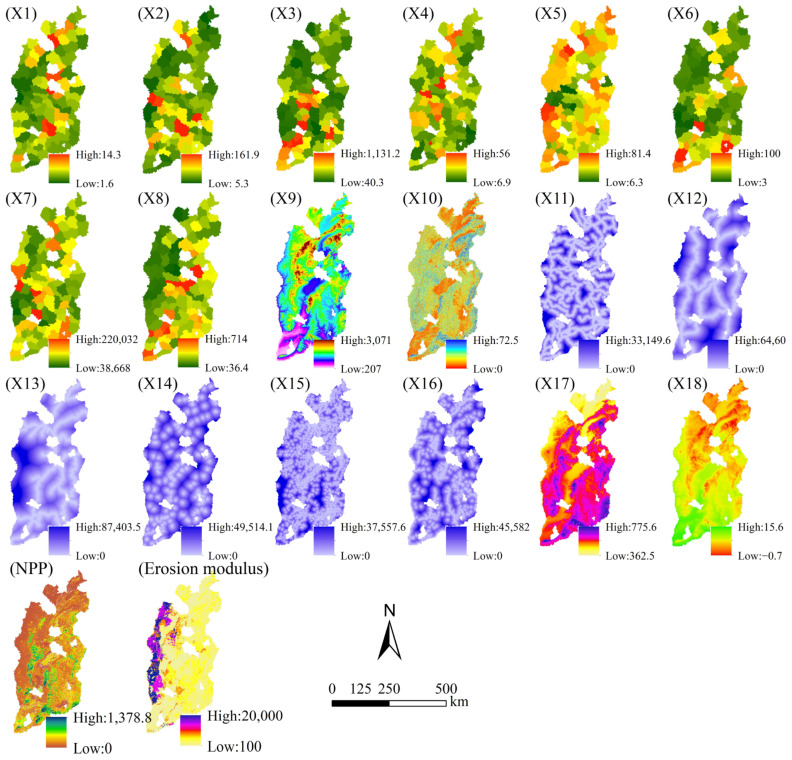
Driving factors of LUCC.

The historical land use data were derived from Landsat TM/ETM images that were generated through visual interpretation based on national field surveys (Table 1). Such data have been previously used in similar studies and exhibit an accuracy of over 90% [4]. The land use data were reclassified into nine types, including cultivated lands, forest lands, orchard lands, grasslands, water bodies, rural construction areas (rural settlements and other construction lands), urban built-up areas, unused land, and wetlands. The distances to urban built-up areas, rural construction areas, and rivers were obtained using ArcGIS reclassification of land use data for each time period that was considered and Euclidean distance calculations at a spatial resolution of 300 m (Figure 2). All the driving force data (Table 1; Figure 2) were resampled to 300 m to reduce the calculation cost and expedite the simulation process.

## 3. Methodologies

### 3.1. Degree of Land Use Dynamism (K)

(1) $$K=\frac{U_{b}-U_{a}}{U_{a}}\times \frac{1}{T}\times 100\%$$

(2) $$S=\left(\overset{n}{\underset{i=1}{\sum }}\frac{\Delta U_{i-j}}{2\sum_{i}^{n}U_{it1}}\right)\times {\left(t_{2}-t_{1}\right)}^{-1}\times 100\%$$

where K is the degree of land use dynamism during the study period; $U_{a}$ and $U_{b}$ are the areas of a land use type at the beginning and end of the study period, respectively; and T is the study period in years; S is the integrated use dynamic attitude of the study area; $\Delta U_{i-j}$ is the area of land type i transformed to non-i land type in the time period $t_{1}-t_{2}$; $U_{it1}$ is the area of land use type i in the study area in time $t_{1}$.

### 3.2. Driving Factors of LUCC and Geographical Detector Model

According to the intensity of LUCC in the study area from 2000 to 2020, four land types with high conversion intensity, namely, cultivated lands, forest lands, grasslands, and rural construction areas, were selected to analyze their driving forces. From four dimensions of population, natural environment, location traffic, and economic development, 18 driving factors (Figure 2) were selected to construct the index system of driving factors. Natural factors include elevation (m), slope (m), distance from river (m), average temperature (°C), and annual average precipitation (mm). Population factor is population density (person/km^2^). Economic factors include per capita value added of primary and secondary industries (10^4^ yuan/person), urbanization rate (%), proportion of agriculture population (%), total power of agricultural machinery (10^4^ kilowatts), local financial expenditures (10^4^ yuan), and grain yield per unit area (kg/ha). There are the following five transportation factors: distance to provincial highway (m), distance to national trunk highway (m), distance to railway (m), distance to rural construction areas (m), and distance to urban construction lands (m). To ensure consistency of data and overcome unit differences among the influencing factors, the 18 selected factors were resampled and classified into 9 levels (slope is classified into 6 levels) according to the natural breakpoint method.

**Figure 3:**
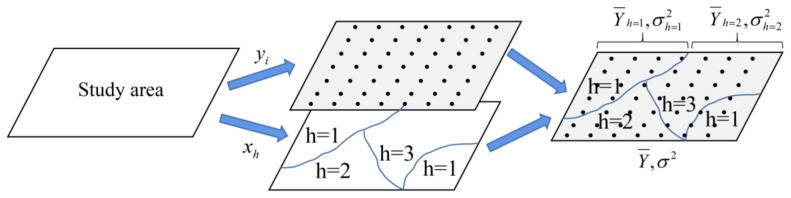
The principle of a geographical detector.

The geographical detector(Figure 3) consists of four detectors [33], namely, factor detector, interaction detector, risk detector, and ecological detector. In this paper, 18 potential driving factors of LUCC in Shanxi were explored by using the factor detector and interaction detector. Factor detector explores the extent to which one factor X (driving factors) explains the spatial differentiation of Y (LUCC) as measured by value q. A larger value of q indicates a greater impact of the factor on LUCC and stronger explanatory power, and conversely, less explanatory power. The expression formula q is as follows:

(3) $$q=1-\frac{\sum_{h=1}^{L}N_{h}\sigma^{2}}{N\sigma^{2}}=1-\frac{SSW}{SST}$$

(4) $$SSW=\overset{L}{\underset{h=1}{\sum }}N_{h}\sigma_{h}^{2}$$

(5) $$SST=N\sigma^{2}$$

where $q\left(0\leq q\leq 1\right)$ is the explanatory power of the influencing factor on LUCC; h is the stratification of variable *Y* or factor *X*; $N_{h}$ and N are the number of stratification samples and the number of cells in the whole area, respectively; $\sigma^{2}$ and $\sigma_{h}^{2}$ are the variance of LUCC in the study area and stratum h, respectively; L is the number of stratifications; SSW and SST are the sum of variance within the stratification and the total variance of the whole area, respectively.

Interaction detector evaluates whether the two influencing factors (X) will increase the explanatory power of the dependent variable Y when they work together. When the two factors interact, the results have the following five situations [31,32]:

(1) $q\left(X1\cap X2\right)\leq Min\left(q\left(X1\right),q\left(X2\right)\right)$, non-linear weakening; (2) $q\left(X1\cap X2\right)>q\left(X1\right)+q\left(X2\right)$, non-linear enhancement; (3) $Min\left(q\left(X1\right),q\left(X2\right)\right)<q\left(X1\cap X2\right)<Max\left(q\left(X1\right),q\left(X2\right)\right)$, single-factor nonlinear weakening; (4) $q\left(X1\cap X2\right)>Max\left(q\left(X1\right),q\left(X2\right)\right)$, two-factor enhancement; (5) $q\left(X1\cap X2\right)=q\left(X1\right)+q\left(X2\right)$, independent.

### 3.3. CA-Markov Model

The expression formulas for the CA-Markov model [16,47] are as follows:

(6) $$S_{\left(t+1\right)}=P_{ij}\times S_{\left(t\right)}$$

where $S_{\left(t\right)}$ and $S_{\left(t+1\right)}$ are the states of the landscape structure at t and t+1 times, respectively, while $P_{ij}$ denotes the transfer state.

The expression formulas for $P_{ij}$ are as follows:

(7) $$P_{ij}=\left[\begin{array}{ccc} P_{11} & \cdots & P_{1n}\\ \vdots & \ddots & \vdots \\ P_{n1} & \cdots & P_{nn} \end{array}\right]$$

where $0\leq P_{ij}<1$, $\overset{n}{\underset{j=1}{\sum }}P_{ij}=1\left(i,j=1,2\cdots ,n\right)$

(8) $$S_{\left(t+1\right)}=f\left(S_{t},N\right)$$

where t and t+1 denote the times before and after the cellular automata, respectively; S is the discrete, finite set of states of the cellular automata; N is the domain of the cellular automata; and f is the spatial transformation rule for the cellular automata.

### 3.4. Scenarios Design

The study designed NG, EP, ED, FS, EP-ED, and EP-FS scenarios to project land use in 2030. The driving factors and weights of the six future scenarios were selected and formulated on the basis of Table 2, and the q values of drivers were standardized as their weights. Under the NG scenario, no policy was involved, and the drivers of LUCC were chosen according to Table 3. Other scenario simulations are based on the NG scenario. Under the EP scenario, the rapid growth of construction land is limited, and the areas with severe soil erosion and low net plant primary productivity (NPP) are protected. The ED scenario is characterized by rapid economic development [48], accelerated urbanization, and encroachment on cultivated lands and grasslands. Under the FS scenario, cultivated lands are prevented from being occupied by built-up areas, and the continuous and stable increase of cultivated land is protected. The EP-ED scenario combines ecological protection and economic development, with ecological protection and economic development as its constraints and drivers, respectively. The EP-FS scenario combines ecological protection and food security, and limits the encroachment of construction lands on cultivated lands and ecological lands.

### 3.5. Model Precision Verification

In the accuracy assessment of LUCC simulation, the sensitivity value (FOM) and kappa coefficient were selected to test the prediction accuracy. The sensitivity value (FOM) index can comprehensively measure the modeling accuracy of simulated changes, focusing on the accuracy of the change area, rather than the accuracy of the entire study area, and is therefore widely used for accuracy verification. The range of FOM is 0–1, where the higher the FOM, the higher the simulation accuracy [49].

(9) $$FOA=\frac{B}{A+B+C+D}$$

where A is the number of cells that changed in the simulated map but actually stayed the same; B is the number of cells that changed in the simulated and actual maps and changed correctly; C is the number of cells that changed in the simulated and actual maps but changed incorrectly; and D is the number of cells that stayed the same in the simulated map but changed in the actual map.

The expression formula for the Kappa Test model is as follows:

(10) $$kappa=\frac{P_{o}-P_{c}}{P_{p}-P_{c}}$$

where $P_{0}$ is the proportion of correct simulations; $P_{c}$ is the expected proportion of correct simulations in random scenarios; $P_{p}$ is the amount of correct simulations in the ideal classification case (i.e., 100%).

**Figure 4:**
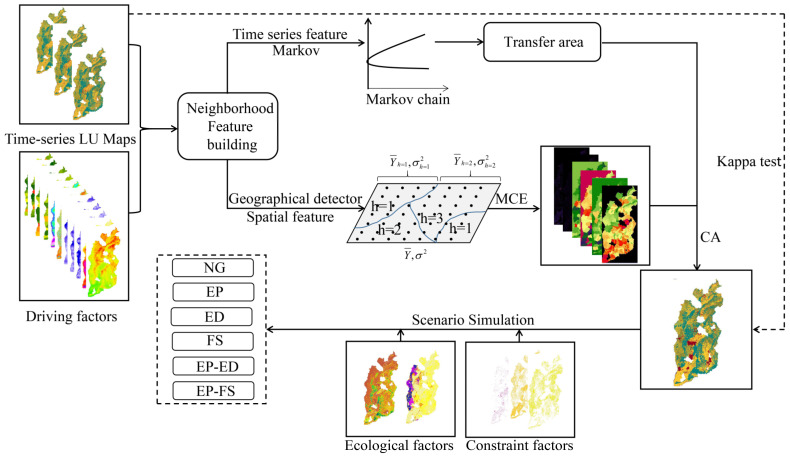
Schematic showing the design framework for this study.

The basic framework design is shown in Figure 4.

## 4. Results and Analyses

### 4.1. LUCC, Transformation Patterns and the Spatial Modes

#### 4.1.1. LUCC

**Figure 5:**
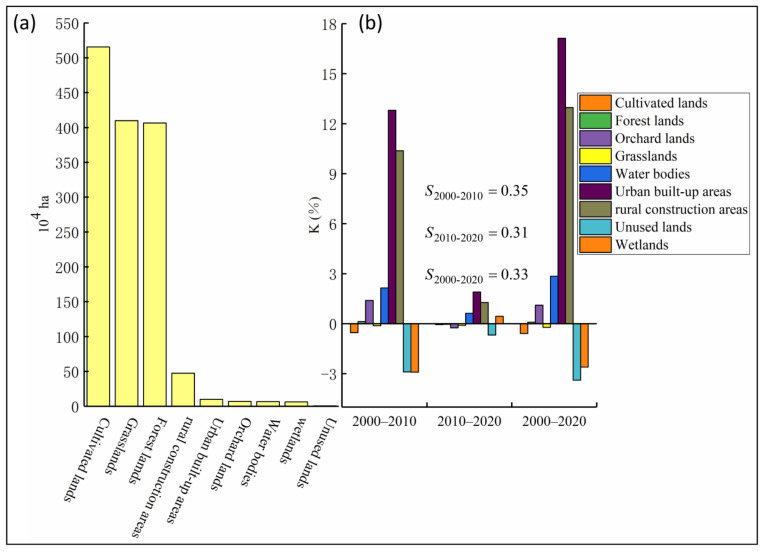
Land use structure (**a**), and dynamic change (**b**).

Using the year 2020 as an illustration (Figure 5a; Table 3), it can be seen that the Shanxi, cultivated lands (36.36%) made up the biggest proportion of land use, followed by grasslands (28.78%), forest lands (28.73%), rural construction areas (3.79%), and urban built-up areas (0.84%).

According to Figure 5b, there was an accelerated change in land use between 2000 and 2010 as the S value was higher than it was between 2010 and 2020. Between 2000 and 2010, the K values for urban built-up areas and rural construction areas were 11.81% and 10.37%, respectively, indicating a strong growth in the area. However, the K values between 2010 and 2020 were 1.9% and 1.27%, respectively, showing a deceleration in the region during that time. Accordingly, the K value for the cultivated land from 2000 to 2010, was −0.53%, showing a significant decrease in croplands throughout this period. Indicating an escalating trend in various land uses, the K values for urban built-up areas, rural construction areas orchard lands, water bodies, and forest lands from 2000 to 2020 were 6.15%, 4.80%, 1.16%, 0.36%, and 0.06%, respectively. Due to their lower size compared to other land use types (Figure 5a), unused lands, orchard lands, wetlands, and water bodies had higher K values.

#### 4.1.2. Land Use Transformation Patterns

The conversion of cultivated lands, grasslands, and forest lands, with contribution values of 17.55 × 10^4^ ha, 5.07 × 10^4^ ha, and 1.64 × 10^4^ ha, respectively, between 2000 and 2010, resulted in an increase in rural construction areas, demonstrating a net increase of 24.15 × 10^4^ ha (or 103.7%) (Table 4); urban built-up areas increased (net increase of 5.56 × 10^4^ ha), which was brought about by the conversion of cultivated lands (3.93 × 10^4^ ha); net increase in forest lands comprised 5.2 × 10^4^ ha, which was primarily brought about by the conversion of cultivated lands (4.19 × 10^4^ ha) and grasslands (2.03 × 10^4^ ha). The rural construction areas increased by a net amount of 6.04 × 10^4^ ha (or 12.7%) between 2010 and 2020, receiving net transfers of cultivated lands, grasslands, and forest lands totaling 3.69 × 10^4^ ha, 1.62 × 10^4^ ha, and 1.4 × 10^4^ ha respectively; the net increase in urban built-up areas was 1.88 × 10^4^ ha, and this primarily occurred due to the conversion of cultivated land (0.97 × 10^4^ ha) and rural construction areas (0.75 × 10^4^ ha).

#### 4.1.3. Land Use Spatial Structure

**Figure 6:**
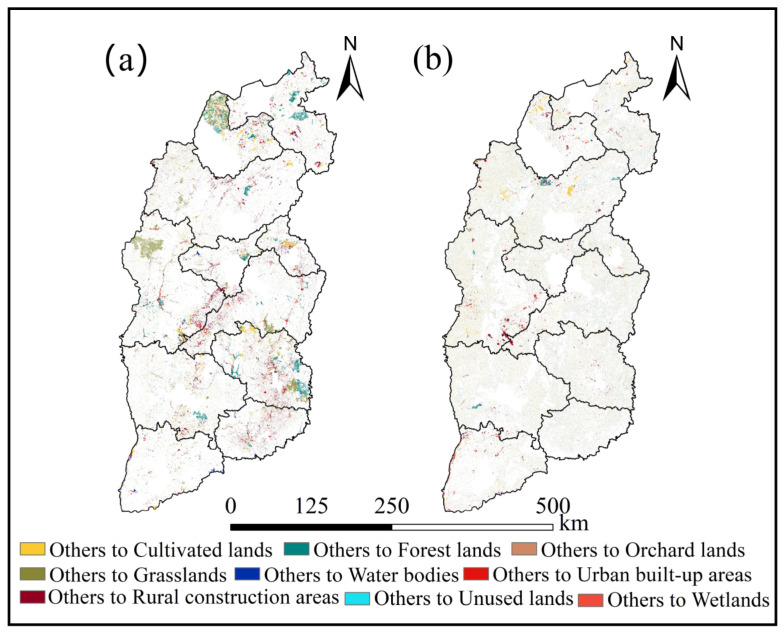
Spatial distribution of LUCCs in Shanxi from 2000–2010 (**a**), and 2010–2020 (**b**).

The northwestern part of Shuozhou City, the core metropolitan region of Taiyuan City, the border region of Lvliang and Jizhong Cities were the main places where LUCC was concentrated between 2000 and 2020 (Figure 6). The central Linfen and Yangquan Cities, as well as the border of Jizhong and Changzhi Cities, saw the greatest growth in cultivated lands between 2000 and 2020. Increases in forest lands were primarily concentrated in the central region of Datong City and the southeastern region of Changzhi City. The core metropolitan areas of Hunyuan and Lingqiu Datong City, Kelan and Wuzhai Counties of Xinzhou City, Jiaocheng County of Lvliang City, and Taiyuan City account for the majority of the growth in the rural construction areas. The increase in grasslands primarily was concentrated in the northwestern region of Lvliang City. Urban built-up areas are concentrated in the Pingcheng and Yungang Counties of Datong City, Yaodu District of Linfen City, as well as the border of Lvliang and Jinzhong City.

### 4.2. Analysis of Drivers of LUCC

#### 4.2.1. Analysis of Single-Factor Drivers

Throughout the study period, the explanatory power of land use drivers displayed diverse characteristics at various stages (Table 2). The conversion of other lands to cultivated lands between 2000 and 2020 was primarily affected by factors such as grain yield per unit area, urbanization rate, population density, local financial expenditure, per capita value added of primary industry, total power of agricultural machinery, precipitation, and grain yield per unit area. In 2000–2010 and 2010–2020, the dominant factors of the increase in forest lands were the proportion of agricultural population (0.57) and the total power of the agricultural machinery (0.40). In addition, from 2000 to 2020, factors such as population density, per capita secondary productivity, per capita primary productivity, urbanization rate, temperature, and elevation played a critical role in the conversion of non-forest lands to forest lands. The conversion of various land use types to grasslands between 2000 and 2020 was significantly influenced by grain yield per unit area, population density, and local financial expenditures. The proportion of agriculture population and the total power of agricultural machinery between 2000 and 2020 had a significantly impact on the conversion of other lands to grasslands. The conversion of other lands to rural construction areas between 2000 and 2010 and between 2010 and 2020 was predominantly impacted by population density and total power of agricultural machinery, with q values being 0.36 and 0.41, respectively. It was also affected by grain yield per unit area, urbanization rate, proportion of agricultural population, per capita added value of secondary industry, and local financial expenditures.

#### 4.2.2. Analysis of Interaction Drivers

**Figure 7:**
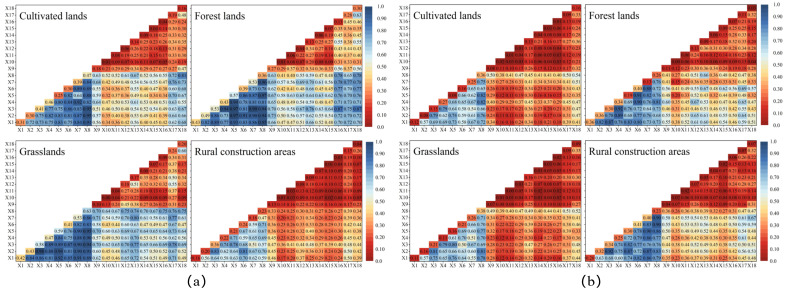
Interaction detection of LUCC drivers from 2000 to 2010 (**a**) and 2010 to 2020 (**b**).

The interactive detection of four drivers of LUCC in Shanxi between 2000 to 2010 and 2010 to 2020 (Figure 7) showed that the interaction of two of the 18 drivers exhibited a nonlinear or synergistic improvement. These results indicated that LUCC was a result of numerous factors, including the natural environment, population, regional, and economic development. Population density, grain yield per unit area, proportion of agriculture population, local finance expenditures, and per capita added value of primary and secondary industries all had relatively strong interactions with LUCC. However, the correlation between LUCC and elevation, slope, precipitation, and temperature were relatively weak. These findings showed that natural factors were the critical basis of the LUCC in Shanxi, while human activities and economics accelerated the development of LUCC.

Grain yield per unit area was only 0.47 for the conversion of other lands to cultivated lands between 2000 and 2010, but the q value for interaction with other factors was above 0.52, and the interaction with population density reached the highest value (0.95); the q value for the interaction between grain yield per unit area and the proportion of agriculture population was the maximum (0.82) during 2010–2020; the average temperature alone has the lowest q value but as it interacts with other factors, the q value rises, reaching a high of 0.72 for the interaction with per capita added of secondary industry. For the conversion of other lands to forest lands, the interaction between population density, local financial expenditures, and the proportion of agriculture population were most significant between 2000 and 2010; urbanization rate, population density, total power of agricultural machinery, and the per capita added value of secondary industry were the highest between 2010 and 2020. As can be seen, the urbanization rate, population density, and economy all have an impact on the conversion of farmlands to forests. For the increase in grasslands, the interaction between per capita value added of secondary industry and population density had the largest q value (0.98) from 2000 to 2010, while the interaction between per capita value added of secondary industry and grain yield per unit area had the highest q value (0.81) from 2010 to 2020. The slope’s impact on the growth of grasslands was the least significant between 2000 and 2020, but it greatly increased after interacting with other factors. Between 2000 and 2020, the effects of per capita value added of secondary industry and population density were most significant for the conversion of other lands to rural construction areas. Interaction with total power of agricultural machinery, proportion of agriculture population, and the local financial expenditures were also higher, which overall reflected the increased demand for built-up areas in the more populated regions.

### 4.3. Simulation of LUCC under Various Scenarios in 2030

The land use for 2020 was predicted using 2000 and 2010, then validated with the actual data from 2020, which revealed a Kappa value of 89.09% and a FOA value of 70.5%. These findings demonstrate the CA-Markov model’s dependably accurate performance for LUCC simulation in Shanxi.

**Figure 8:**
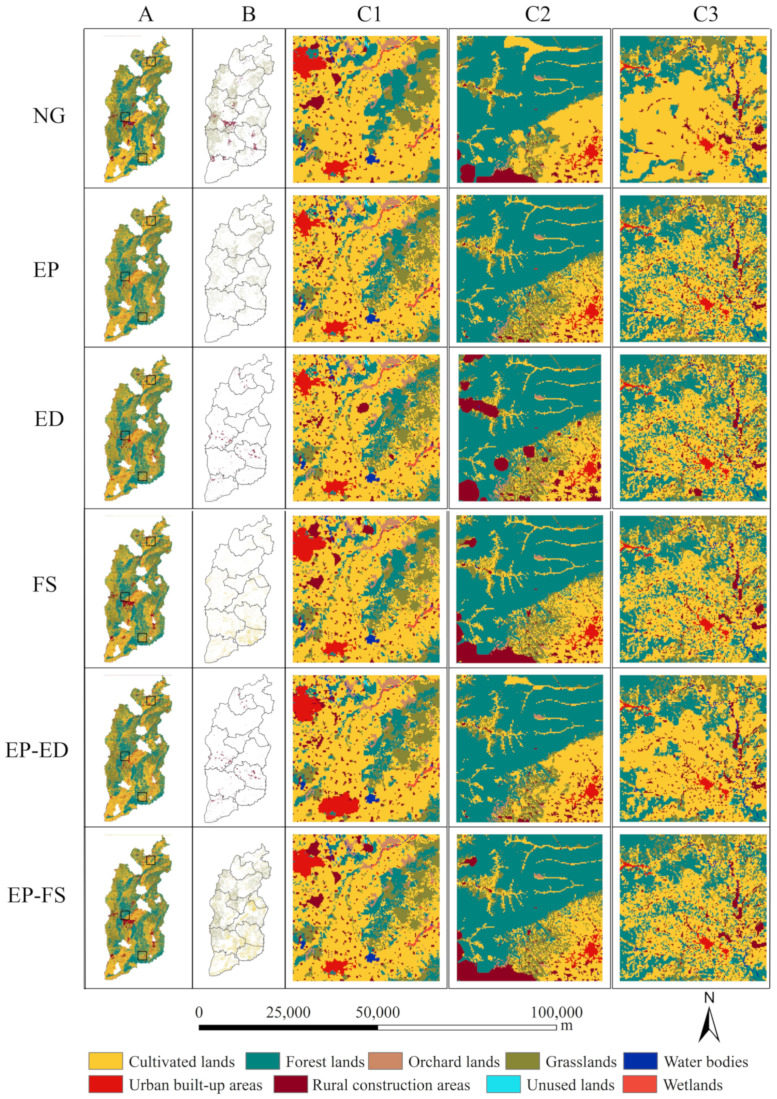
Spatial distribution of land use in Shanxi under the six scenarios estimated for 2030. (**A**,**B**) indicate the simulation results of land use in 2030 and the spatial distribution of area increase of each land use type, respectively; (**C1**–**C3**) indicate the three randomly selected areas from (**A**).

Figure 8A shows the spatial distribution of land use in Shanxi under the six scenarios estimated for 2030. We arbitrarily chose three regions for comparison in order to further explain the inconsistent findings of land use modeling under different scenarios (Figure 8C1–C3). The conversion of forest lands under the NG scenario resulted in an increase in grasslands and construction lands (Table 5). The distribution of grasslands increased, but it was dispersed, with the majority of it concentrated in the southeast of Lvliang and Linfen Cities, in the Lingqiu County of Datong City, and in the Youyu and Jingle Counties of Shuozhou City (Figure 8B). The construction land was primarily concentrated in the surrounding region of Taiyuan, Fenyang, and Changzhi Cities, the junction of Xiaoyi and Jiexiu Cities, and Linfen and Yuncheng Cities. According to the EP scenario, the conversion of grasslands into cultivated lands was primarily concentrated in Lingqiu County of Datong City, Wutai and Jingle County of Qizhou City, and Yushe County of Jizhong City. The conversions to rural construction areas dominate the LUCC processes in the ED and EP-ED scenarios, and the growth harms largely the cultivated lands and grasslands. The increase in rural construction areas was primarily concentrated in Fenyang City, Xiaoyi City, and Lucheng County. Under the scenario of FS, the increase in cultivated land primarily occurred due to the conversion of grasslands and forest lands, and which were particularly concentrated in the south of Shanxi, the Jizhong City, and its fertile soil region. According to the scenario of EP-FS, both cultivated lands and grasslands increased fragmentally, with cultivated lands being primarily concentrated in Shanxi’s southeast, and grasslands being primarily concentrated in its northeast and west.

## 5. Discussion

### 5.1. Spatiotemporal LUCC Characteristics

Socio-economic development and rapid urban expansion have exacerbated the tension between humans and the environment [23,50]. According to the survey, Shanxi’s construction areas both showed a substantial increased trend between 2000 and 2020. Rural construction areas increased primarily derived from conversions of cultivated lands and grasslands, and they were primarily concentrated in the core metropolitan area of each county, such as Hunyuan and Lingqiu Counties, Bali and Wuzhai Counties, Jiaocheng County, and the center of Taiyuan and Datong City. The increase of urban built-up areas was primarily derived from conversions of cultivated lands, and this was mostly concentered around the area of the core metropolitan. These findings agreed with those of other researchers [51,52] who have examined local locations in China. Economic benefits are often the primary consideration in the management of land resource in developing countries [53]. The GDP of Shanxi increased more than nine times between 2000 and 2020, and improvements in financial conditions have prompted a sizable influx of people from rural to urban areas, promoting the growth of cities and surrounding built-up areas. Changes in perceptions and levels of consumption have also led to structural changes in agriculture, such as the conversion of some cultivated lands to orchard lands. Between 2000 and 2020, forest lands were primarily derived from conversions of cultivated land and grasslands, and this was linked to specific policies promulgated by the government, such as returning farmland to forests [54]. The main goals of the policy are to preserve high-quality croplands, convert cultivated lands vulnerable to soil erosion and desertification, convert parts of the steppe to become forest lands of high ecosystem service values, and restore forest vegetation. These changes have resulted in significant changes in land use in the study areas, especially in areas with intensive agricultural activities. This has resulted in a gradual decrease in cultivated lands and grasslands, mainly concentrated in the center of Datong City and the southeastern part of Changzhi City. Policies and regulations contribute the most to LUCC, resulting in the fragmentation of land structure and food security [55,56,57]. As the world’s most populous country, improving the efficiency of the use of cultivated lands and ensuring food security is of critical significance. Strengthening farmland protection is the most important mechanism for stabilizing the domestic food supply. Therefore, Shanxi should establish an effective land use mechanism, control urban expansion, balance urban construction land and rural construction areas [58], increase investment in agricultural water conservancy facilities [59], and strictly protect high-quality cultivated lands.

### 5.2. The Response of Driving Factors to LUCC

LUCC results from the interplay of multiple factors, which play a role at different spatiotemporal scales [25]. This paper explores the driving mechanisms of changes in cultivated lands, forest lands, grasslands, and rural construction areas from the dimension of the natural environment, population, location and transportation, and economic development using a geographic probe model. The theoretical universality and practical applicability remain to be further tested. The interaction detection results of the driving factors of the four LUCCs showed that the interactions of the selected 18 factors exhibited a two-factor enhancement or a non-linear enhancement. The dominant factor in the increase of cultivated lands from 2000 to 2020 was grain yield per unit area. However, Arowolo and Deng [60] studied the drivers of cropland change in Nigeria and the results showed biophysical, socio-economic, and proximity factors are significant determinants of transition to cultivated land use. The results differ from those of this paper, which may be related to the different drivers selected. The crop production environment and land production capacity are reflected by grain yield per unit area, which determined the growing conditions of the regional soil environment [61,62]. Therefore, the cultivated land is more likely to transfer to areas with fertile soils. In addition to demographic and economic impacts, forest lands are strongly influenced by natural factors (temperature and elevation). Forest lands can provide animals with enough water, food, habitat, breeding areas, and other essential environmental conditions [62,63]. Thus, a continuous and stable growth of forest lands should be maintained in the future, which will lead to a more stable ecological structure. Grain yield per unit area, population density, and local financial expenditures were the major driving forces in grasslands. Grasslands played a significant role value in the development of animal husbandry [64] and the protection of biodiversity [65], the conservation of soil and water [66], and ecological balance. Areas with poor soils and low population fall more readily into wasteland and thus naturally develop into weed lands. However, financial expenditures for ecological conservation can protect natural grasslands and prevent their degradation. The increase in rural construction areas was mostly influenced by population density and total power of agricultural machinery in 2000–2010 and 2010–2020, respectively. Rapid economic development and increased agrarian machinery have left a surplus of rural labor. In addition, engaging in non-agricultural activities increased the net income per capita of rural residents, resulting in farmers’ reluctance to rely on their own farmland for subsistence and thus migrate to towns for high returns [67,68]. The migration of populations to cities accelerates the demand for construction land and promotes the expansion of rural residential land around cities.

### 5.3. Future Land Use Scenario Simulation and Deficiencies

Extensive field surveys are difficult and costly [69]. In contrast, satellite remote sensing may be the only economically viable method to routinely collect data on land types within large watersheds at high spatiotemporal resolutions [70,71,72]. The land use remote sensing data in this paper are based on remotely sensed imagery from Landsat TM satellites in the U.S. The accuracy of Landsat generated by national field surveys and manual visual interpretation was greater than 90% cent [73]. For this paper, we first checked the accuracy of the forecast simulations and found that the Kappa value of both the valid and predicted values for 2020 was 89.09%, and the FOA value was 70.1%. The simulation performance of these models is generally acceptable. Six scenarios of land use in 2030 depicting different development strategies were designed to consider socio-economic development, ecological protection, and food security. The EP scenario limits the expansion of built-up areas and protects ecologically vulnerable areas, and these simulation results are reflected in the increase of grasslands. The NG, ED, and EP-ED scenarios accelerate urbanization, which leads to a significant increase in built-up areas, and these observations are also reflected in our study. The FS and EP-FS scenarios protect high quality cultivated lands, resulting in a continuous increase in farmland area. All these simulation results are clearly consistent with our expected results. Therefore, the simulation results of this paper for different scenarios of land use in 2030 have high reliability and can truly reflect the land use results under different scenarios.

Grasslands increase sporadically in scenarios of NG, EP, and EP-FS. Increases in grasslands are linked to changes in socio-economic factors, such as the marginalization of cultivated lands and the loss of labor due to the rise in the opportunity cost of farming, which in turn leads to the abandonment of cropland. Grasslands contribute to regional ecology not only by increasing carbon sequestration and oxygen release [74,75], but also by controlling regional temperature and moisture through vegetation transpiration, thereby regulating the climate [76,77]. As a result, political strategies such as “Ecological Red Line” should be implemented to protect ecological land and gradually improve the quality of the environment [78]. Furthermore, implementing ecological protection should focus on extremely ecologically sensitive regions (such as hilly mountainous areas or regions prone to soil erosion), and public service positions should be set up to protect ecological achievements. Eco-agriculture and special cultivation will be vigorously developed in highly ecologically sensitive areas to enhance the ecological habitat environment. For the cultivated lands that are difficult to transport and abandon, emphasis should be on encouraging forest planting and giving subsidies. Built-up areas under scenarios of NG, ED, and EP-ED increase substantially through encroachment on cultivated lands, grasslands, and forest lands. In the future, we should coordinate urban land planning and ecosystem pattern optimization, encourage the revitalization of stock construction land, steadily reduce the scale of new construction land, improve the land management system, avoid the waste of land caused by rapid land urbanization, and circumvent the adverse effects of rapid land urbanization. In both the FS and EP-FS scenarios, cultivated lands are increasing. China, as the world’s most populous agricultural country, faces enormous resource and environmental pressures and economic and social risks in food production on the assumption that the potential for developing domestic farm resources is approaching its limit. We should improve the strict cultivated land protection system and the system of saving and intensive use. Cultivated lands remain the basis of the food supply. Ensuring sufficient cultivated lands and steadily increasing the grain production capacity of cropland are fundamental to ensuring the country’s food security. Future land use scenarios are beneficial for decision-makers to make good decisions that balance socio-economic, ecological, and food security benefits.

Despite the excellent performance of the Geodetector-MCE-CA-Markov model in land use simulation, there are still some gaps in the study due to limitations in data collection and the research methods. First, for temperature, precipitation, NPP, and erosion modulus data due to the availability of their resources, the average data from 2005 to 2015 were used instead of the average data from 2000 to 2020 in this paper. This may have a small error in the analysis of the results. Further, it was not precise to determine what the impact factors are for each land use type. Finally, some critical parameters, such as EP and FS, are neglected in the simulations. Ecological protection should consider carbon storage which is also an essential parameter for measuring the ecological environment [79,80]. The simulation of FS scenarios should take into account regional differences in climate factors, cropping systems, agricultural development, land transfer, and socio-economic development through extensive field research and farmer surveys. Future work should therefore consider the impact of policies on land drivers across multiple scenarios in an integrated way. The mechanisms of LUCC should be further explored through a combination of more comprehensive and more accurate data to select directions for land use optimization.

## 6. Conclusions

The structure, spatiotemporal changes, and transformation characteristics of LUCC in Shanxi from 2000 to 2020 were analyzed, and the driving mechanism for changes in cultivated lands, forest lands, grasslands, and rural construction areas was analyzed using the geographical detector from four dimensions, namely, population, natural environment, location traffic, and economic development. Simulated parameters were set based on the above findings, and the CA-Markov model was used to simulate the land use under different scenarios in 2030. The results indicated that: (1) Rural construction areas, urban built-up areas, water bodies, forest land and orchards showed an increasing trend from 2000 to 2020, while cultivated lands, grassland, unused land, and wetland showed a decreasing trend. The increase in rural construction areas and forest lands occurred due to the transformation of cultivated land and grasslands, and the increase in urban built-up areas and orchards primarily occurred due to the conversion of cultivated land. (2) The increase of cultivated lands, forest lands, grasslands, and rural construction areas was primarily influenced by the amount of grain yield per unit area, urbanization rate, population density, and local financial expenditures, and both two-factor interactions showed a nonlinear or synergistic enhancement. The synthesis indicated that the increase in cultivated lands, forest lands, grasslands, and rural construction areas were constrained by natural factors and driven by population and economic development as a population–economic–natural dominated driver. (3) Under the NG, ED, and EP-ED scenarios, the built-up areas will increase significantly, while under the FS and EP-FS scenarios, the cultivated lands will increase. These future land use scenarios can inform decision-makers to make sound decisions that balance socio-economic, ecological, and food security benefits.

## Figures and Tables

**Table 1:** Data format and sources.

Dataset	Format	Resolution	Time	Data Source
Land use/cover	Raster	30 m	2000, 2010, 2020	Data Center for Resources and Environmental Sciences of the Chinese Academy of Sciences (http://www.resdc.cn/ (accessed on 20 June 2021))
Temperature	Raster	1 km	2005–2015	
Precipitation	Raster	1 km	2005–2015	
NPP	Raster	1 km	2005–2015	
Erosion modulus	Raster	1 km	2005–2015	
Roads	Vector	30 m	2020	OpenStreeMap (https://www.openhistoricalmap.org/ (accessed on 20 June 2021))
Population density	Excel	-	2000–2020	Chinese National Bureau of Statistics (http://www.stats.gov.cn/ (accessed on 20 June 2021))
Economic indicators				
Elevation	Raster	30 m	2020	Data Center of Geospatial data cloud (https://www.gscloud.cn/ (accessed on 20 June 2021))
Slope	Raster	30 m	2020	

**Table 2:** Explanatory forces of LUCC drivers during 2010–2020 (q ).

	Land Use Types	X1	X2	X3	X4	X5	X6	X7	X8	X9	X10	X11	X12	X13	X14	X15	X16	X17	X18
2000 to 2010	Cultivated lands	0.31	0.30	0.41	0.46	0.25	0.30	0.39	0.47	0.18	0.01	0.08	0.06	0.14	0.09	0.04	0.04	0.19	0.16
	Forest lands	0.43	0.49	0.53	0.43	0.57	0.39	0.53	0.36	0.27	0.01	0.06	0.04	0.13	0.06	0.07	0.13	0.28	0.30
	Grasslands	0.42	0.43	0.58	0.47	0.59	0.41	0.53	0.63	0.10	0.00	0.04	0.18	0.17	0.08	0.07	0.09	0.24	0.20
	Rural construction areas	0.16	0.20	0.36	0.22	0.21	0.24	0.18	0.23	0.13	0.01	0.03	0.08	0.09	0.07	0.01	0.03	0.15	0.04
2010 to 2020	Cultivated lands	0.12	0.08	0.15	0.27	0.08	0.16	0.25	0.36	0.08	0.02	0.01	0.01	0.13	0.04	0.03	0.01	0.09	0.16
	Forest lands	0.36	0.36	0.30	0.34	0.19	0.40	0.13	0.26	0.11	0.00	0.06	0.13	0.09	0.06	0.09	0.07	0.11	0.03
	Grasslands	0.11	0.16	0.21	0.11	0.16	0.21	0.26	0.38	0.07	0.01	0.00	0.03	0.16	0.01	0.03	0.02	0.09	0.09
	Rural construction areas	0.20	0.32	0.34	0.25	0.30	0.41	0.40	0.23	0.04	0.02	0.02	0.07	0.05	0.04	0.02	0.06	0.07	0.07

Note: X1, X2, X3, X4, X5, X6, X7, X8, and X9 indicate per capita value added of primary industry, per capita value added of secondary industry, population density, urbanization rate, proportion of agriculture population, total power of agricultural machinery, local financial expenditures, grain yield per unit area, elevation (m), and slope; X10, X11, X12, X13, X14, X15, and X16, indicate distances to provincial highway, national trunk highway, railway, urban construction lands, rural construction areas, and river; X17 and X18 indicate average temperature and annual average precipitation.

**Table 3:** NG scenario parameter setting (J-shape curve).

Land Use Types	Monotonically Increasing	Monotonically Decreasing
Cultivated lands	Grain yield per unit area, total power of agricultural machinery, precipitation, urbanization rate, per capita value added primary industry, distance from railway, proportion of agriculture population	Elevation
Forest lands	Per capita value added of primary industry, elevation, local financial expenditures	Grain yield per unit area, distance from national trunk highway
Grasslands	Elevation	population density, grain yield per unit area, average annual precipitation, proportion of agriculture population, total power of agricultural machinery
Rural construction areas	Urbanization rate, population density, local financial expenditures, per capita value of added secondary industry	Proportion of agriculture population, elevation

**Table 4:** Land use conversion area (10^4^ ha).

	Land Use Types	Cultivated Lands	Forest Lands	Orchard Lands	Grasslands	Water Bodies	Urban Built-Up Areas	Rural Construction Areas	Unused Lands	Wetlands	Total
2000 to 2010	Cultivated lands	496.79	7.68	1.84	14.23	1.16	4.05	18.55	0.05	0.34	544.70
	Forest lands	3.49	387.73	0.31	7.79	0.18	0.13	1.74	0.02	0.03	401.41
	Orchard lands	0.32	1.01	4.60	0.10	0.01	0.03	0.11	0.00	0.01	6.17
	Grasslands	11.51	9.82	0.21	387.01	0.29	0.49	5.32	0.06	0.10	414.81
	Water bodies	0.57	0.10	0.01	0.17	3.97	0.08	0.26	0.06	0.36	5.58
	Urban built-up areas	0.12	0.01	0.00	0.01	0.00	4.07	0.13	0.00	0.00	4.34
	Rural construction areas	1.00	0.10	0.01	0.25	0.07	0.99	20.86	0.00	0.01	23.29
	Unused lands	0.29	0.04	0.02	0.10	0.00	0.00	0.07	0.44	0.01	0.96
	Wetlands	1.45	0.13	0.04	0.21	1.09	0.05	0.41	0.04	5.45	8.87
	Total	515.55	406.61	7.03	409.87	6.77	9.90	47.44	0.68	0.34	1410.14
2010 to 2020	Cultivated lands	484.15	5.41	0.16	16.13	0.75	1.31	6.95	0.03	0.76	515.67
	Forest lands	6.43	390.55	0.24	7.41	0.06	0.09	1.70	0.02	0.05	406.55
	Orchard lands	0.42	0.10	6.32	0.10	0.01	0.00	0.08	0.00	0.01	7.03
	Grasslands	17.27	8.60	0.10	380.96	0.20	0.14	2.33	0.04	0.18	409.82
	Water bodies	0.31	0.06	0.00	0.18	5.58	0.01	0.09	0.04	0.48	6.75
	Urban built-up areas	0.34	0.01	0.00	0.07	0.00	9.33	0.11	0.00	0.03	9.90
	Rural construction areas	3.26	0.30	0.02	0.71	0.06	0.86	42.17	0.00	0.05	47.44
	Unused lands	0.06	0.01	0.00	0.02	0.06	0.00	0.01	0.48	0.04	0.68
	wetlands	0.49	0.08	0.01	0.21	0.45	0.03	0.03	0.03	4.96	6.29
	Total	512.73	405.13	6.86	405.79	7.17	11.78	53.48	0.64	6.56	1410.14

**Table 5:** The area and proportion of land use types under various scenarios from 2000 to 2030.

Year	Area (10^4^ ha)	Cultivated Lands	Forest Lands	Orchard Lands	Grasslands	Water Bodies	Urban Built-Up Areas	Rural Construction Areas	Unused Lands	Wetlands
2000		542.72	401.41	6.17	414.81	5.58	4.34	25.42	0.96	8.87
2010		515.70	406.61	7.03	409.87	6.77	9.90	47.44	0.68	6.29
2020		512.77	405.10	6.86	405.79	7.29	11.78	53.49	0.64	6.58
2030	NG	512.05	383.05	6.37	413.14	6.89	14.51	66.76	0.59	6.92
	EP	501.26	399.25	6.79	423.21	7.59	13.17	51.70	0.68	6.65
	ED	502.05	405.62	6.72	400.37	7.13	13.06	68.08	0.64	6.62
	FS	531.87	397.81	6.51	396.37	6.89	14.15	49.52	0.69	6.49
	EP-ED	502.08	405.57	6.72	400.40	7.12	13.06	68.08	0.64	6.62
	EP-FS	521.39	386.80	6.46	415.70	6.93	14.20	51.36	0.61	6.84

## Data Availability

The datasets used and/or analyzed during the current study are available from the corresponding author upon reasonable request and the approval of the data owner.
